# Human urinary kallidinogenase may improve the prognosis of acute stroke patients with early neurological deterioration

**DOI:** 10.1002/brb3.2524

**Published:** 2022-02-13

**Authors:** Yiju Xie, Shengyu Li, Jian Zhang, Shijian Chen, Xuhui Deng, Gengyu Cen, Zhijian Liang

**Affiliations:** ^1^ Department of Neurology The First Affiliated Hospital of Guangxi Medical University Nanning China; ^2^ Department of Neurology Wuming Hospital of Guangxi Medical University Nanning China; ^3^ Department of Neurology The Second Affiliated Hospital of Guangxi Medical University Nanning China

**Keywords:** acute ischemic stroke, early neurological deterioration, human urinary kallidinogenase, intravenous thrombolysis, prognosis

## Abstract

**Objectives:**

Some acute ischemic stroke (AIS) patients still suffer from early neurological deterioration (END) after receiving intravenous thrombolysis (IVT), and these patients often have a poor prognosis. The purpose of our study is to observe the efficacy and safety of human urinary kallidinogenase (HUK) treatment in patients with END.

**Methods:**

This was a retrospective analysis and 49 patients with END who met the inclusion criteria were divided into the observation group and the control group. All patients received routine treatment of AIS, while patients in the observation group were treated with HUK within 24 h after IVT and the other group without HUK.

**Results:**

There were 24 patients in the observation group and 25 patients in the control group. After treatment, favorable prognosis (mRS scores ≤2) at 3 months in the observation group with 13 cases (54.17%) was significantly better than that in the control group with four cases (16%) (*p* = .001), and there was no statistical difference between the two groups in any hemorrhagic complication.

**Conclusion:**

HUK is considered to be safe and may improve the prognosis of AIS patients with END after IVT. More clinical trials are needed to validate these results in the future.

## Graphical abstract

Acute cerebral infarction patients with early neurological deterioration often have a poor prognosis. Human urinary kallidinogenase may be a possible treatment to improve the outcome of them.

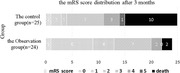

## 1 INTRODUCTION

Intravenous thrombolysis (IVT) or combined bridging therapy is the most effective treatment recommended by current guidelines for acute ischemic stroke (AIS) patients (Powers et al., 2019). However, it was reported that 3.0%–32.8% of acute stroke patients suffered from early neurological deterioration (END) within 24 h after IVT which often greatly increases the risk of disability and mortality (Y.‐L. Liu, Yin, et al., 2020; Marto et al., 2019). Apart from straightforward causes, such as intracerebral hemorrhage (ICH) and malignant edema, the mechanism of END after IVT remains unclear in most instances (Yu et al., 2020). Therefore, how to improve the prognosis of patients with END after IVT has become a clinical problem that urgently needs to be addressed.

The ARTIS trial (Antiplatelet Therapy in Combination With recombinant tissue plasminogen activator Thrombolysis in Ischemic Stroke) found that early application of intravenous aspirin in patients with AIS after IVT does not improve the clinical outcome at 3 months but increases the risk of symptomatic intracranial hemorrhage (sICH) (Zinkstok et al., 2010). Nevertheless, a study recently got a different conclusion that early antithrombotic therapy might be a feasible treatment to solve the dilemma (Tisserand et al., 2014). Then, a retrospective clinical research found that a low dose of tirofiban in patients with END within the first 24 h after IVT did not increase the risk of sICH, ICH as well as mortality, and seemed to be associated with neurological improvement at 3 months (C. Wu et al., 2019). On the whole, the fact that whether the usage of antiplatelet agents within the first 24 h after IVT increased the risk of sICH still needs to be validated. Meanwhile, a systematic review and meta‐analysis recommended that a good collateral circulation was closely related to the favorable outcome, and it was suggested to develop therapeutic approaches to augment collateral perfusion as an adjunctive strategy for acute stroke treatment (Leng et al., 2016).

Human urinary kallidinogenase (HUK), a tissue kallikrein, was demonstrated to augment collateral perfusion in animal experiments and patients with AIS (L. Han et al., 2015; Miao et al., 2016). It was recommended by Chinese Guidelines to treat the patients with acute ischemic stroke in 2018 (Level Ⅱ recommendation, Level B evidence) (Qian et al., 2019). Based on the appellate theory, clinicians try to apply HUK to the treatment of stroke patients who develop END after IVT. The present study was conducted to observe the efficacy and safety of HUK in the treatment in the patients with END after IVT with tissue‐type plasminogen activator (rt‐PA) by retrospectively analyzing the basic data of AIS patients from three comprehensive stroke centers.

## 2 METHODS

### 2.1 Study population

This study was a multicenter retrospective study. From January 1, 2019 to December 31, 2020, a total of 302 AIS patients who received IVT from three comprehensive stroke centers (including the First Affiliated Hospital of Guangxi Medical University, the Second Affiliated Hospital of Guangxi Medical University, and the Wuming Hospital of Guangxi Medical University) were selected as study population. END was defined as an increase of ≥4 points in the baseline National Institutes of Health Stroke Scale (NIHSS) score or an increase of more than 2 points in the NIHSS score over the same subcategory when it deteriorated within 24 h (Leng et al., 2016). Fifty‐eight patients received IVT developed END, and nine cases were excluded for already known causes of deterioration. Forty‐nine patients with END were finally enrolled and divided into the observation group (24 patients) and the control group (25 patients). Patients in both groups were given routine treatment of acute cerebral infarction (neuroprotective therapy, anti‐platelet therapy, statins, etc.). Among them, antiplatelet drugs were administered 24 h after IVT by excluding contraindications. All patients with END were timely reexamined by head computed tomographic, magnetic resonance imaging, computed tomographic angiography, or other imaging examinations to prove no signs of ICH or malignant edema. HUK was added to the conventional treatment in the observation group within 24 h after IVT, while the control group was treated without HUK during hospitalization and within 3 months after discharge.

#### 2.1.1 Inclusion criteria

The inclusion criteria were as follows:

1. the standard of IVT with rt‐PA was met in the International Guidelines for the Early Management of Acute Ischemic Stroke, and the dose of rt‐PA was calculated according to the standard dose of 0.9 mg/kg (Powers et al., 2019);
2. age ≥18 years old;
3. NIHSS score of stroke patients ≤25 points;
4. END occurred within 24 h after IVT.

#### 2.1.2 Exclusion criteria

The exclusion criteria were as follows:

1. consistent with contraindications for IVT;
2. the standard of rt‐PA intravenous thrombolysis was met but the total dose of rt‐PA was less than 0.9 mg/kg;
3. patients who took any anticoagulant drugs in 1 week before stroke onset;
4. NIHSS score of patients ≥25 points;
5. patients who met END but received endovascular treatment such as mechanical thrombectomy, stent implantation, and artery thrombolysis;
6. patients who met END but deteriorated due to sICH, malignant edema, early recurrent ischemic stroke, and the poststroke seizure.

#### 2.1.3 Standard of treatment

#### Intravenous thrombolytic therapy

The applied dose of rt‐PA is the standard dose (0.9 mg/kg). The maximum dose is not more than 90 mg. Initially, 10% of the total dose was given in the first phase, and the remaining dose was infused intravenously within 1 h.

#### Treatment of HUK

The observation group received HUK treatment within 24 h after IVT. The drug was named human urinary kallidinogenase for injection (0.15 pNA, Chinese medicine approval H20052065, Guangdong Tianpu Biochemical and Pharmaceutical company). Note that 0.15 pNA unit drug was dissolved in 100 ml sodium chloride and injected intravenously for 30 min once a day. The course of treatment was 10–14 days.

#### 2.1.4 Collection indicators

Baseline demographic and clinical information of all enrolled patients was collected from medical records and national stroke databases, including name, gender, age, related risk factors of stroke (including hypertension, diabetes, coronary heart disease [CHD], atrial fibrillation [AF], smoking history, and previous use of anticoagulant drugs in 1 week before stroke), baseline NIHSS score at admission and when it developed into END, modified Rankin Scale (mRS) at admission and the one at 3 months, systolic blood pressure (SBP) at the time of IVT and when it deteriorated, random blood glucose before IVT, the TOAST classification of cerebral infarction, and laboratory indexes (including the levels of triglyceride [TG], total cholesterol [TC], low‐density cholesterol [LDL], high‐density lipoprotein [HDL], and homocysteine [HCY] at admission). There are three calculate indicators including body mass index (BMI), the time from the onset of stroke to IVT (t1), and the time from the end of IVT to the onset of deterioration (t2).

#### 2.1.5 Assessment method

#### Classification of stroke

Stroke is classified according to the TOAST classification system (Adams et al., 1993), including large‐artery atherosclerosis (L‐type), small‐artery atherosclerosis (S‐type), cardiogenic cerebral embolism (C‐type), other determined etiology (O‐type), and undetermined causes (U‐type).

#### Definition of END

END can be defined as an increase of ≥4 points in the baseline NIHSS score or an increase of more than 2 points in the NIHSS score over the same subcategory when it deteriorated within 24 h (C. Wu et al., 2019).

##### The efficacy outcomes

The efficacy outcomes of HUK are based on the mRS score at 3 months. The prognosis of the patients with END is divided into poor (mRS score >2 or death) and favorable levels (mRS score ≤2).

##### Safety assessment

The safety outcomes included the incidence of sICH, ICH, and systemic hemorrhage and mortality at 3 months. sICH is defined according to ECASS III, in which the increase of 4 points in the patient's NIHSS score from baseline or a direct result of death is because of intracranial hemorrhage associated with worsening clinical symptoms (Yaghi et al., 2017). The classification standard of intracranial hemorrhage was based on the Heidelberg Hemorrhage Classification in 2015 (Von Kummer et al., 2015). Among them, severe systemic bleeding is defined as life‐threatening bleeding that needs immediate medical intervention.

## 3 STATISTICAL ANALYSIS

Data processing and statistical analysis were performed using the SPSS 20.0 software. The normality of data distribution was assessed by Shapiro–Wilk normality test. The continuous variables are described as average ± standard deviation (X ± S) or median (interquartile range). The *t*‐test or Mann–Whitney *U* test was applied in the intergroup comparison. Categorical variables are presented as absolute and relative frequencies. Pearson *χ* ^2^ test correction for continuity or Fisher's exact test was used for intergroup comparison. Binary logistic regression analysis was used to evaluate the effect of HUK on the prognosis of patients with END. For multivariate logistic regression analysis, we included any confounding variables with *p* < .1 and other selected baseline characteristics. *p* < .05 on both sides was considered statistically significant.

## 4 RESULTS

**FIGURE 1:**
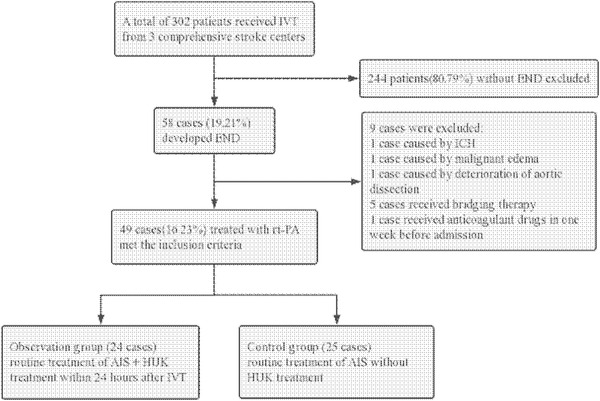
Flow chart of enrolled patients

A total of 302 patients with AIS received IVT, and 58 patients (19.21%) developed END. Nine cases were excluded for already known causes of deterioration so far, including one case caused by intracranial hemorrhage, one case caused by malignant edema, one case who developed END due to low perfusion caused by aortic dissection in subsequent examination after IVT, five cases received bridging treatment after deterioration, and one case took anticoagulant drugs in 7 days before admission. In the end, 49 patients with END treated with rt‐PA met the criteria of this study (the inclusion process is shown in Figure 1).

Of all the enrolled END 49 patients, 24 patients (the observation group) chose HUK treatment within the first 24 h after IVT, while the remaining 25 patients (the control group) did not. The comparison of the demographic and baseline data between the two groups is shown in Table 1. The ages of the control group were older than the observation group with statistically significant (*p* = .04). For the related risk factors of cerebral infarction, hypertension was the most common risk factor accounting for 71.43%, followed by smoking, diabetes, AF, and CHD. There was no statistical difference between the risk factors mentioned above between the groups. The average baselines NIHSS scores were evenly distributed, but the NIHSS scores on deterioration of the control group were statistically higher than that of the observation group (12 vs. 10) (*p* = .04). When it comes to the TOAST classification of stroke, although there was no statistical difference in TOAST classification between groups, the proportion of C‐type in the control group was higher than that in the observation group. t1 was 2.91 ± 0.92 (h) and t2 was 10.57 ± 7.47 (h) on average. The comparison of t1 and t2 between the two groups was not statistically significant, but the patients in the control group developed END earlier than that in the observation group (8.64 h vs. 12.49 h). In short, the results showed that there was no statistical difference in all indicators but the age and NIHSS scores at the time of deterioration.

**TABLE 1:** Comparison of the demographic and baseline data between the two groups

Variable	All of END 49 cases	Observation group 24 cases (48.98%)	Control group 25 cases (51.02%)	*p*
Age (year)	62.92 ± 11.75	59.42 ± 12.83	66.28 ± 9.72	.04
Gender (male)	30 (61.22%)	15 (62.5%)	15 (60%)	.86

Related risk factors of stroke				
Hypertension	35 (71.43%)	16 (66.67%)	17 (68%)	.92
Diabetes	11 (22.45%)	5 (20.83%)	6 (24%)	.73
CHD	3 (6.12%)	2 (8.33%)	1 (4%)	.97
AF	9 (18.37%)	2 (8.33%)	7 (28%)	.14
Smoking history	17 (34.69%)	8 (33.33%)	9 (36%)	.85
Antiplatelet drugs use in 1 week before stroke	7 (14.29%)	4 (16.67%)	3 (12%)	.95

Clinical indicators at admission				
SBP at baseline	156.80 ± 19.89	159.83 ± 18.45	153.88 ± 21.14	.30
Glucose	6.2 (5.4–7.5)	6.3 (5.5–7.8)	5.8 (5.3–7.4)	.50
Baseline NIHSS	8 (4–12)	8.5 (4–12)	8 (4–12)	.80

Indicators on deterioration				
SBP on deterioration	139.96 ± 21.45	139.50 ± 21.90	140.40 ± 21.46	.89
NIHSS on deterioration	11 (8–18)	10 (7–13)	12 (8–25)	.04

Laboratory indicators				
TC	4.42 ± 1.05	4.60 ± 0.68	4.25 ± 1.31	.25
TG	1.03 (0.75–1.42)	1.02 (0.79–1.42)	1.06 (0.63–1.42)	.52
LDL	2.90 ± 0.82	2.93 ± 0.70	2.87 ± 0.93	.25
HDL	1.12 (0.95–13.2)	1.12 (0.96–1.33)	1.14 (0.95–1.31)	.96
HCY	13.12 (10.00–14.69)	12.79 (9.97–15.03)	13.20 (10.04–14.65)	1

TOAST classification				
L‐type	28 (57.14%)	15 (62.5%)	13 (52%)	.46
C‐type	9 (18.37%)	2 (8.33%)	7 (28%)	.14
S‐type	10 (20.41%)	5 (20.83%)	5 (20%)	1
O‐type	1 (2.04%)	1 (4.16%)	0	.49
U‐type	1 (2.04%)	1 (4.16%)	0	.49

Calculated indicators				
BMI	23.71 ± 2.93	23.97 ± 3.24	23.45 ± 2.65	.55
t1 (hour)	2.91 ± 0.92	2.96 ± 0.90	2.85 ± 0.96	.68
t2 (hour)	10.57 ± 7.47	12.49 ± 6.66	8.64 ± 7.69	.12

*Note*: t1, the time from the onset of stroke to IVT; t2, the time from the end of IVT to the onset of deterioration. L‐type, large‐artery atherosclerosis; S‐type, small‐artery atherosclerosis; C‐type, cardiogenic cerebral embolism; O‐type, other determined etiology; U‐type, undetermined causes.

Abbreviations: AF, atrial fibrillation; BMI, body mass index; CHD, coronary heart disease; END, early neurological deterioration; HCY, homocysteine; HDL, high‐density lipoprotein; LDL, low‐density cholesterol; NIHSS, National Institutes of Health Stroke Scale; SBP, systolic blood pressure; TC, total cholesterol; TG, triglyceride.

The safety outcomes included the incidence of sICH, ICH and systemic hemorrhage, and the mortality at 3 months. No sICH or systemic hemorrhage occurred in the observation group during the treatment process, while there was one case of sICH and one of systemic bleeding, respectively, in the control group. The complication systemic hemorrhage was characterized by gastrointestinal bleeding. There were three patients (12.5%) of hemorrhage transformation in the observation group and one patient (4%) in the control group. The mortality within 3 months in the control group with 10 cases (40%) was significantly higher than that in the observation group with two cases (8.33%). Pearson *χ* ^2^ test results showed that there was no significant difference in the complications of sICH, ICH, and systemic hemorrhage between the groups (Table 2).

**TABLE 2:** Safety and effectiveness analysis

Safety indicators	All of END49 cases	Observation group24 cases	Control group25 cases	*p*
SICH	1 (2.04%)	0	1 (4%)	1
All ICH	5 (10.20%)	3 (12.5%)	2 (8%)	.96
Severe systemic bleeding	1 (2.04%)	0	1 (4%)	1
Mortality of 3 m	12 (24.49%)	2 (8.33%)	10 (40%)	.01

Efficacy outcomes				
Favorable prognosis	17 (34.69%)	13 (54.17%)	4 (16%)	.001

Abbreviations: END, early neurological deterioration; ICH, intracranial hemorrhage; SICH, symptomatic intracranial hemorrhage.

**FIGURE 2:**
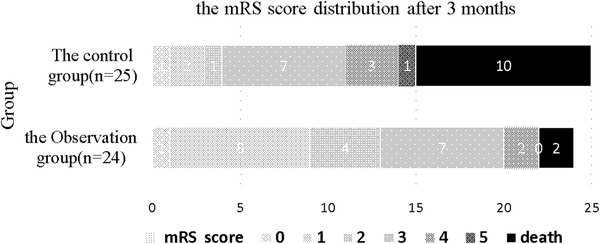
mRS distribution between the two groups after 3 months

The efficacy outcomes of HUK are based on the mRS score at 3 months (the distribution between groups is shown in Figure 2). At 3 months, the favorable prognosis of the observation group with 13 cases (54.17%) is higher than that of the control group with four cases (16%) (*p* = .001). Binary logistic regression analysis was used to analyze the influence of HUK on the prognosis of patients with END. The results of univariate and multivariate logistic regression analyses of the prognosis of patients with END are shown in Tables 3 and 4, respectively. The outcome of *p*‐values and odds ratio (OR) before and after adjusting age, atrial fibrillation, high‐density lipoprotein, and NIHSS scores on deterioration was .002 versus .024 and 0.10 versus 0.14, respectively, as shown in Table 4.

**TABLE 3:** Univariate logistic regression analysis of the prognosis of early neurological deterioration (END) patients

Variable	*p*
Age(year)	.030
Gender (male)	.330
Hypertension	.725
Diabetes	.559
CHD	.959
AF	.096
Smoking history	.949
Antiplatelet drugs use in 1 week before stroke	.626
SBP at baseline	.137
Glucose	.731
Baseline NIHSS	.123
SBP on deterioration	.387
NIHSS on deterioration	.027
TC	.222
TG	.179
LDL	.661
HDL	.096
HCY	.109
L‐type	1.000
C‐type	1.000
S‐type	1.000
O‐type	1.000
U‐type	1.000
BMI	.150
t1 (hour)	.624
t2 (hour)	.856
HUK treatment	.002

*Note*: t1, the time from the onset of stroke to IVT; t2, the time from the end of IVT to the onset of deterioration; L‐type, large‐artery atherosclerosis; S‐type, small‐artery atherosclerosis; C‐type, cardiogenic cerebral embolism; O‐type, other determined etiology; U‐type, undetermined causes.

Abbreviations: AF, atrial fibrillation; BMI, body mass index; CHD, coronary heart disease; HCY, homocysteine; HDL, high‐density lipoprotein; LDL, low‐density cholesterol; NIHSS, National Institutes of Health Stroke Scale; SBP, systolic blood pressure; TC, total cholesterol; TG, triglyceride.

**TABLE 4:** Multivariate logistic regression analysis of the effect of human urinary kallidinogenase (HUK) on the prognosis of early neurological deterioration (END) patients

	HUK treatment		Unadjusted analysis		Adjusted analysis^a^	
	Yes (*n* = 24)	No (*n* = 2 5)	OR (95% CI)	*p*	OR (95% CI)	*p*
Favorable prognosis	13 (54.17%)	4 (16%)	0.10 (0.02–0.42)	.002	0.14 (0.03–0.77)	.024

Abbreviations: CI, confidence interval; OR, odds ratio.

^a^ After adjusting age, atrial fibrillation, high‐density lipoprotein, and NIHSS score at deterioration.

## 5 DISCUSSION

In present study, the incidence of END was 19.21%. Previous studies found that it is not uncommon for patients with ACI after IVT to develop END. In terms of incidence, a meta‐analysis found that the rate of END after IVT was 11.0% globally and varied by region, with a highest incidence of 15.9% in Asia, 11.8% in North America, and 7.6% in Europe (Hou et al., 2019). The incidence of END in present study was close to that in Asia in the meta‐analysis.

In present study, it was found that use of HUK within the first 24 h after IVT improved the prognosis of patients with END significantly and did not increase the risk of sICH, ICH, and systemic bleeding. In fact, END has been paid more and more attention because of its unfavorable prognosis. Attempts to improve the outcome of patients with END have been ongoing for many years. Therapies such as usage of antiplatelet drugs tirofiban after IVT had been found to improve the outcome of patients with END (B. Liu, Zhang, et al., 2020). However, whether antiplatelet drugs within 24 h after IVT will increase the risk of intracranial hemorrhage remains to be further demonstrated. Many studies have demonstrated the safety of using HUK in patients with AIS. C. Li, Zhao, et al. (2015) treated AIS patients with different TOAST types with HUK and found that cerebral hemorrhage occurred in only one patient with cardiogenic cerebral infarction of 110 patients. A meta‐analysis on the use of HUK to patients with AIS including 16 trials with 1326 participant found that when compared with the control group, there were no reports of death or any severe adverse reactions, such as cerebral hemorrhage, in all studies (Huang et al., 2020).

There is one innovation in our research. Most of the studies about the treatment of HUK were done on general patients with AIS, while our study's population was aimed at the special part of them, the ones with early neurological deterioration after intravenous thrombolysis. Studies have shown that treatment with HUK can reduce the rate of disability of stroke patients (Dong et al., 2020; D. Han et al., 2018; D. Wu et al., 2017). In our study, 54.17% of patients with END treated with HUK recovered well. Although the sample size in our study is not large enough, however, considering the nature of our study is a registration one, the data were retrospectively collected and the findings of our research were derived from the real world. The findings are useful for clinicians to make decisions for AIS patients who received IVT to some extent.

Furthermore, in what way HUK improves the prognosis of END is an interesting question. First, it was found that HUK could activate the kalinase system, selectively dilate the microvessels in the ischemia regions, and promote the formation of revascularization in the ischemic area (L. Han et al., 2015). Second, HUK can selectively dilate infarcted blood vessels, increase blood flow of ischemic areas, and improve the nerve function of patients by improving microcirculation (Sun et al., 2016). Third, an animal experiment showed that HUK also had a certain function of anti‐inflammation, anti‐apoptosis, and promoting neurogenesis (L. Han et al., 2015). Among these mechanisms, the “collateral circulation mechanism” is relatively recognized for now. Cerebral collateral circulation varies greatly among individuals, and a good collateral circulation is often associated with a good long‐term neurological function for stroke patients after receiving IVT (Wang et al., 2015). A Chinese study applying magnetic resonance perfusion imaging to examine patients with ACI treated with HUK for 12 days demonstrated that blood flow increased in ischemic area and was associated with prognosis improving, suggesting that HUK augmented collateral perfusion (J. Li, Chen, et al., 2015). Hence, we speculate that HUK may improve the prognosis of patients with END by promoting the formation of collateral circulation and enlarging collateral perfusion.

Our study has some limitations. First, it is a retrospective study with a small sample. Second, the collateral state of patients with END was not evaluated before and after the usage of HUK. As a result, how HUK improves the prognosis of patients with END remains unclear.

## 6 CONCLUSIONS

The present study suggests that HUK administration within the first 24 h after IVT with rt‐PA may not increase the risk of sICH, ICH, and systemic bleeding, and it may improve the prognosis of the patients who developed early neurological deterioration, but that needs more large and prospective studies to prove.

## CONFLICT OF INTEREST

The authors declare no conflict of interest.

## AUTHOR CONTRIBUTIONS

Yiju Xie, Shengyu Li, and Zhijian Liang conceived and designed the experiments. Gengyu Cen, Yiju Xie, Shengyu Li, and Jing Zhang helped to collect the data. Yiju Xie, Shijian Chen, and Xuhui Deng analyzed the data and performed the statistical analysis. Jian Zhang and Zhijian Liang provided critical strategic advice. Zhijian Liang provided financial support for this work. Yiju Xie collected the data and wrote the manuscript. All authors read and approved the final manuscript.

### PEER REVIEW

The peer review history for this article is available at https://publons.com/publon/10.1002/brb3.2524.

## Data Availability

All data generated or analyzed during this study are included in this article.
